# Association of Toll-Like Receptor 4 Gene Polymorphisms with Acute Aortic Dissection in a Chinese Han Population

**DOI:** 10.1155/2020/8306903

**Published:** 2020-12-09

**Authors:** Tan Li, Xiaozheng Liu, Hongxia Ning, Xintong Li, Jun Yang, Chunyan Ma

**Affiliations:** ^1^Department of Cardiovascular Ultrasound, The First Hospital of China Medical University, Shenyang 110001, China; ^2^Department of Vascular and Thyroid Surgery, The First Hospital of China Medical University, Shenyang 110001, China

## Abstract

**Background:**

Inflammation may be involved in the pathogenesis of acute aortic dissection (AAD). Toll-like receptor 4 (*TLR4*) is known to play a critical role in regulating the immune and inflammatory processes. To date, the relationship between genetic variation of *TLR4* and AAD is far from clear. The purpose of our study was to illustrate the relevance of *TLR4* polymorphisms with the susceptibility to AAD.

**Methods:**

A total of 222 AAD patients and 222 controls were enrolled in this study. Frequency distributions of *TLR4* polymorphisms (rs10759932 in the promoter and rs11536889 in the 3′-untranslated region) were determined by the KASP method. Clinical parameters were acquired from subjects' medical records, and serum *TLR4* levels were collected from our previously published data.

**Results:**

We found that rs10759932 polymorphism was associated with a reduced risk of AAD in the overall population (CC vs. TT: OR = 0.393, 95%CI = 0.164‐0.939, *P* = 0.036; recessive model: OR = 0.439, 95%CI = 0.196‐0.984, *P* = 0.045) and subgroup analyses stratified by sex. The GC genotype and dominant model of rs11536889 conferred a significantly higher risk of AAD compared with GG genotype in female subjects (GC vs. GG: OR = 3.382, 95%CI = 1.051‐10.885, *P* = 0.041; dominant model: OR = 3.043, 95%CI = 1.041‐8.900, *P* = 0.042). In addition, a significant interaction between the rs11536889 recessive model and dyslipidemia was observed for an increased risk of AAD (*P*_interaction_ = 0.038, OR = 15.229) after the adjustment for potential clinical covariates. We also used the false-positive report probability (FPRP) analysis to validate the significant results. Furthermore, rs11536889 polymorphism could affect the maximal aortic diameters of AAD (*P* = 0.037), while AAD patients carrying CC genotype of rs10759932 showed lower serum *TLR4* levels than TT genotype carriers (*P* = 0.043).

**Conclusions:**

Our findings provide evidence for the association between *TLR4* polymorphisms and AAD susceptibility in a Chinese Han population, which may have some implications for understanding the role of *TLR4* in the pathophysiology of AAD.

## 1. Introduction

Acute aortic dissection (AAD) is the most life-threatening macrovascular disorder with high mortality, characterized by intimal tear and surging of blood into the medial layer of the aorta [1, 2]. The etiology of AAD is complex and heterogeneous. In addition to some environmental risk factors, such as smoking, male, old age, hypertension, and dyslipidemia, genetic factors are also viewed to contribute to the pathogenesis of AAD [3, 4]. Although the responsible molecular and genetic determinants of AAD remain largely unidentified, it has been widely accepted that inflammation plays an essential role in the structural damage of the aortic wall and the development of AAD [2, 5, 6]. Increasing data indicated that elevated inflammatory cell infiltration and higher expression levels of inflammatory mediators, including C-reactive protein (CRP) and D-dimer, were detected in dissected aortic specimens and peripheral blood of AAD patients [7–9]. More importantly, the local inflammation in the aortic wall and subsequent systemic inflammatory reaction were observed during the whole course of AAD [2, 10, 11]. As a consequence, investigating the inflammation-related pathogenic genes would be beneficial to understand the underlying mechanisms of AAD and to prevent and treat the disease.

Toll-like receptor 4 (*TLR4*), located on chromosome 9q32-q33, is not only the key pattern-recognition receptor in immune-inflammatory reactions but also the initiation protein in this signal transduction pathway [12]. Recently, the role of *TLR4*-mediated signaling has emerged in maintaining aortic homeostasis and establishing the aorta alterations (vascular remodeling and medial degeneration) and their complications [13, 14]. It was confirmed that the upregulation of *TLR4* could evocate inflammatory cell infiltration, production of proinflammatory mediators, endothelial dysfunction, smooth muscle cell apoptosis, and aortic media degradation, which were closely related to aortic inflammation, remodeling, and dissection [15–17]. In our previous study, we preliminarily found that an elevated level of serum *TLR4* expression was independently associated with the risk of AAD, and there was a positive relationship between serum *TLR4* and circulating CRP [18]. The activity and function of *TLR4* seem to be modulated by genetic variations, principally single nucleotide polymorphisms (SNPs), which may change the ligand binding and balance between pro- and anti-inflammatory cytokines, thereby regulating the progression of various diseases [19, 20]. Therefore, it is reasonable to hypothesize that SNPs in the functional regions of the *TLR4* gene may have effects on *TLR4* activity and thus modify the signaling of immune and subsequent inflammatory responses, which in turn may affect AAD risk. Some evidences have exhibited that *TLR4* polymorphisms were closely associated with the risk of infection [21], atherosclerotic disease [22], autoimmune disease [23], and tumor [24]. One study revealed that polymorphisms linked to the *TLR4*-mediated metalloproteinase pathways could obviously impact the risk of sporadic thoracic aortic aneurysm [25]. It remains unclear, however, whether *TLR4* polymorphisms are relevant to the susceptibility of AAD.

In this case-control study, we aimed to discuss the correlation between *TLR4* polymorphisms and AAD risk and examine whether potential gene-environment interactions could enhance the susceptibility to AAD. In addition, the consequences of these SNPs on the levels of AAD-related parameters and serum *TLR4* in AAD patients were further investigated. Our study might contribute to the prediction of genetic variants associated with disease risk and add knowledge for the prevention and treatment for AAD.

## 2. Material and Methods

### 2.1. Study Population

A total of 222 AAD patients and 222 controls were enrolled from the First Hospital of China Medical University from May 2017 to August 2018. Case and control participants were all Chinese Han population and matched by age and sex. All the patients were evaluated within 24 hours after symptom and diagnosed by the computed tomography angiography (CTA), and there were 172 patients under surgical treatment. Excluding criteria incorporated the subjects with coronary heart diseases, congenital cardiovascular defects, severe vascular stenosis, autoimmune diseases, severe organ failure, infectious diseases, hematological system diseases, or malignant tumors. Patients with certain genetic syndromes, such as Marfan syndrome and Ehlers-Danlos syndrome, or traumatic aneurysms were also excluded from the study. A 5 mL fasting venous blood sample was obtained from each subject for DNA isolation. The study was approved by the Ethics Committee of the First Hospital of China Medical University (Shenyang, China). Written informed consent was obtained from each participant.

### 2.2. Data Collection

The demographic data and clinical related information were collected from participants' medical records. And maximal aortic diameters of AAD subjects were assessed by CTA. Smoking was defined as having smoked at least one cigarette per day for more than one year. Drinking was defined as having consumed at least one alcoholic drink a day for a minimum period of six months. Body mass index (BMI) was computed as weight in kilograms divided by the square of height in meters. Obesity was defined as BMI ≥ 28 kg/m^2^. Hypertension was defined as systolic blood pressure (SBP) ≥ 140 mmHg and/or diastolic blood pressure (DBP) ≥ 90 mmHg and/or use of antihypertensive medications. Diabetes was defined as fasting plasma glucose (FPG) ≥ 7 mmol/L (126 mg/dL) and/or under antidiabetic treatment. Dyslipidemia was defined as total cholesterol (TC) ≥ 6.22 mmol/L (240 mg/dL), or triglyceride (TG) ≥ 2.26 mmol/L (200 mg/dL), or high-density lipoprotein cholesterol (HDL‐C) < 1.03 mmol/L (40 mg/dL), or low-density lipoprotein cholesterol (LDL‐C) ≥ 4.14 mmol/L (160 mg/dL) and/or under taking hypolipidemic drugs.

### 2.3. SNP Selection and Genotyping Assay

A two-step approach was adopted to identify tag-SNPs in *TLR4* [26]. Briefly, we applied the combination of HapMap database (http://www.HapMap.org) and Haploview software 4.2 (http://www.broadinstitute.org/mpg/haploview) to select the tag-SNPs, which should fit the following criteria: minor allele frequency (MAF) > 0.05 in Chinese Han population, low linkage disequilibrium (*r*^2^ < 0.8), and Hardy-Weinberg equilibrium (HWE) > 0.05. Then, potential functions of tag-SNPs were predicted with SNPinfo Web Server (https://snpinfo.niehs.nih.gov/). Accordingly, rs10759932 in the promoter region and rs11536889 in the 3′-untranslated region (3′-UTR) of *TLR4*, which could separately modify the function of transcription factor binding sites and miRNA binding sites, were chosen in this study.

A routine phenol-chloroform method was utilized to extract genomic DNA from each blood clot. All samples were randomly placed on the 384-well plates and blinded for disease status. SNPs were genotyped by Baygene Biotechnology Company Limited (Shanghai, China) with the KASP method using the SNPLine platform (LGC, United Kingdom). Genotyping quality was assessed by repeated detection of 10% randomly selected samples, yielding a 100% concordance.

### 2.4. SNP-Gene Expression Correlation Analysis

Based on our previously published data [18], a total of 64 AAD patients with the information of serum *TLR4* levels were involved in further genotype and *TLR4* gene expression correlation analysis.

### 2.5. Statistical Analysis

All the data analyses were conducted with SPSS 17.0 software (SPSS Inc., Chicago, IL, United States). HWE for studied SNPs in each group was evaluated with the chi-square (*χ*^2^) test. Differences of baseline characteristics between AAD patients and controls were compared by the independent-sample *t*-test or *χ*^2^ test as appropriate. Comparisons of continuous variables among different genotype groups were performed with one-way ANOVA. The association of SNPs with AAD risk was estimated by calculating odds ratios (ORs) and their 95% confidence intervals (CIs) using multivariate logistic regression after adjusting the potential confounding factors. The log-likelihood ratio test was performed to evaluate the SNP-environment interaction by comparing the model that only involved the main effects with the full model also containing the interaction term. The Bonferroni correction was used to adjust *P* values for multiple tests as needed. Moreover, the false-positive report probability (FPRP) was calculated to verify the significant results at different prior probability levels. First, we used the software NCSS-PASS version 11.0.7 (USA) to test the statistical power of each association. Then, the FPRP values were figured out by following the published instructions, and only the significant result with FPRP < 0.5 was regarded as a noteworthy finding [27]. A two-sided *P* < 0.05 was considered statistically significant. In addition, the dominant and recessive genetic models were defined as heterozygote+homozygote variant vs. homozygote wild and homozygote variant vs. heterozygote+homozygote wild, respectively.

## 3. Results

### 3.1. Characteristics of the Study Population

Table 1 presents the baseline characteristics of the study participants. Compared with controls, AAD cases were not statistically different in age, sex, obesity, smoking, drinking, and dyslipidemia.

### 3.2. Association of TLR4 Polymorphisms with AAD Risk

The genotype distributions of rs10759932 and rs11536889 in each group are summarized in Table 2. The genotypes in controls were all in consistent with HWE (*P* > 0.05). After adjusting age, sex, obesity, smoking, drinking, hypertension, diabetes, and dyslipidemia, the rs10759932 CC genotype and recessive model were associated with a decreased risk of AAD with corresponding ORs of 0.393 (95%CI = 0.164‐0.939, *P* = 0.036) and 0.439 (95%CI = 0.196‐0.984, *P* = 0.045), respectively. The overall genetic effects for rs11536889 related to AAD were not observed.

To explore the correlation between *TLR4* polymorphisms and AAD risk in specific subgroups, we further conducted stratified analyses on the basis of age and sex, as shown in Table 3. For rs10759932, the recessive model was associated with a reduced AAD risk in male subjects (OR = 0.343, 95%CI = 0.133‐0.882, *P* = 0.026), and the heterozygote TC and dominant model conferred a decreased risk of AAD in female subjects (TC vs. TT: OR = 0.231, 95%CI = 0.071‐0.752, *P* = 0.015; dominant model: OR = 0.241, 95%CI = 0.082‐0.707, *P* = 0.010). As for rs11536889, its GC genotype and dominant model were significantly correlated with an increased risk of AAD in female subjects with OR values of 3.382 and 3.043 (all *P* < 0.05), respectively, compared with GG genotype.

### 3.3. The Interactions between TLR4 Polymorphisms and Risk Factors in AAD Susceptibility

The interaction effect between *TLR4* polymorphisms and environmental factors on the risk of AAD was examined. A combined genotype including the dominant and recessive genetic models of *TLR4* SNPs was used for interaction analysis.  Table 4 showed that the most significant interaction was between the rs11536889 recessive model and dyslipidemia and associated with an increased risk of AAD (*P*_interaction_ = 0.038, OR = 15.229), after the adjustment for age, sex, obesity, smoking, drinking, hypertension, and diabetes. However, there were no significant interactions between rs10759932 and risk factors in AAD susceptibility.

### 3.4. FPRP Results

Now that AAD is a relatively rare disease and there are limited studies concerning the association between gene polymorphism and AAD risk, we set 0.5 as the FPRP threshold [27]. It was shown that all of the significant findings for *TLR4* rs11536889 polymorphism remained noteworthy at the prior probability of 0.25 or 0.1 (Table 5).

### 3.5. The Association of TLR4 Polymorphisms with Clinical Parameters and Serum TLR4 Levels in AAD Patients

**Figure 1:**
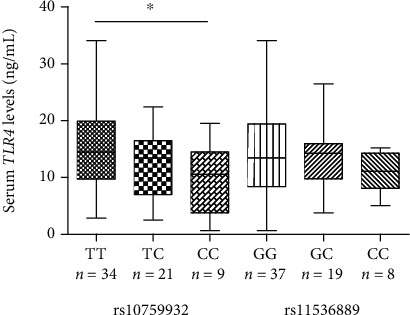
The effect of *TLR4* rs10759932 and rs11536889 polymorphisms on serum *TLR4* levels. ^∗^*P* < 0.05.

Larger aortic diameters were observed in rs11536889 CC genotype carriers when compared to GG genotype carriers (*P* = 0.037) (Table 6). In addition, AAD subjects with CC genotype had significantly lower serum *TLR4* levels than those with TT genotype for *TLR4* rs10759932 (*P* = 0.043) (Table 6 and Figure 1).

## 4. Discussion

To our knowledge, no investigation has focused on the association between *TLR4* polymorphisms and AAD susceptibility. The current study is the first report to identify the significance of *TLR4* rs10759932 and rs11536889 polymorphisms and their interactions with environmental factors in the risk of AAD, as well as their associations with AAD-related clinical parameters and serum *TLR4* levels.

AAD is one of the severe and major health issues of aortic disease known to be caused by inflammation, which could destroy the aortic structure and eventually lead to the aortic wall dissection and rupture [28]. *TLR4* is considered a useful marker for evaluating local inflammatory reaction and has attracted particular interest because of its important function in mediating vascular remodeling and injury [15, 17]. As the most common form of genetic variation, SNPs in *TLR4* functional regions might cause a dysfunction of *TLR4* molecule and interfere with the host immunity and inflammation response, contributing to the risk of various diseases. The SNP rs10759932 locates in the promoter region of the *TLR4* gene and may regulate the *TLR4* expression level by influencing the binding affinity of transcription factors [29]. The T to C allele substitution of rs10759932 has been reported to be strongly associated with a reduced risk of malignant tumors [29, 30]. Similarly, our results indicated a significant association of rs10759932 CC genotype and recessive model with a reduced risk of AAD in the overall population, and the favorable effect of rs10759932 polymorphism on AAD was also prominent in the subgroup analyses stratified by sex. The rs11536889 polymorphism is located in the centre of the 2818-bp *TLR4* 3′-UTR, where a genetic change can influence mRNA stability and translation efficiency [24, 31]. A laboratory study by Sato et al. revealed that a fragment of 3′-UTR containing the *TLR4* rs11536889 G allele, but not the C allele, inhibited luciferase activity triggered by LPS or IL-6 possibly by binding to miRNAs in posttranscriptional regulation [32]. Several studies found that *TLR4* rs11536889 CC genotype or C allele was correlated with an increased risk of coronary artery disease [12], gastric cancer [33], and hepatitis A virus infection [34]. In this research, we figured out that female individuals carrying rs11536889 GC genotype or dominant model were more susceptible to AAD compared with those with GG genotype. The above findings implied that *TLR4* rs10759932 and rs11536889 could be genetic biomarkers and potential therapeutic targets for AAD.

AAD is a complex trait, and its susceptibility may be enhanced by a combined effect of genetic background and environmental exposure [5]. We further analyzed the interaction of *TLR4* rs10759932 and rs11536889 polymorphism with obesity, smoking, drinking, hypertension, and dyslipidemia in the risk of AAD. Only the effect between the rs11536889 recessive model and dyslipidemia was observed to relate to an increased risk of AAD with an OR value of 15.229 after adjusting the potential confounders. Abnormal blood lipid composition has been demonstrated to play a key role in the occurrence and development of aortic dissection [35]. Moreover, as endogenous ligands, modified lipoproteins may activate the *TLR4* signaling pathway and influence the function of *TLR4* [36, 37]. Recent evidence suggested that hyperlipidemia was able to modulate *TLR4* activation and contribute to the increased *TLR4* expression [38, 39]. Our findings indicated that the genetic variation along with dyslipidemia could lead to a synergistic reaction on AAD susceptibility.

Activation of *TLR4* can induce the production and secretion of proinflammatory cytokine, while AAD is frequently accompanied by a systemic inflammatory response and severe coagulopathies, which may be provoked by acute aortic injury and reflected in an increment in serum levels of WBC count, CRP, and D-dimer [8, 40, 41]. The maximal aortic diameter is a well-established determinant of adverse clinical events in patients with aortic disorder. The combined application of genetic analysis and noninvasive vascular imaging holds promise for the prediction and risk stratification of AAD patients [42]. Interestingly, although our results showed no significant association of *TLR4* rs10759932 or rs11536889 polymorphism with serum WBC, CRP, and D-dimer levels, AAD patients with rs11536889 CC genotype displayed larger aortic diameters compared to those with GG genotype. Furtherly, our genotype-phenotype analysis suggested that the CC genotype of rs10759932 linked with AAD susceptibility contributed to lower serum *TLR4* levels than the TT genotype. Thus, we speculate that rs10759932 polymorphism could have a protective effect on AAD risk by downregulating the expression of *TLR4*. However, further molecular experiments should be carried out to verify our results.

Several limitations existed in the current study. First, because of the fact that AAD was a rare vascular disease, our sample size was relatively small, especially for stratification and interaction analyses, so further replication and validation in larger and different populations were required. Second, there were some missing data in demographic and clinical parameters. In addition, our investigation was a genetic association study and lacked in vitro functional confirmation tests. Therefore, a series of experiments were needed to identify the potential molecular mechanisms of the significant SNPs in the future.

## 5. Conclusion

In summary, our data demonstrated that *TLR4* rs10759932 was a protective factor whereas rs11536889 was a risk factor for AAD in a Chinese Han population, and these genetic correlations were independent of the classical cardiovascular risk factors. And the interaction between rs11536889 recessive model and dyslipidemia could enhance the susceptibility to AAD. Furthermore, rs11536889 had a significant impact on AAD size, and rs10759932 was in an evident association with serum *TLR4* expression levels. Our findings may provide context for the better understanding of genetic features of AAD and thus facilitate the improvement of diagnostic and therapeutic approaches for AAD patients.

## Figures and Tables

**Table 1:** Baseline characteristics of the study participants^a^.

Variables	Control (*n* = 222)	AAD (*n* = 222)	*P*
Age (years)	56.7 ± 11.8	56.3 ± 12.2	0.769
Male, *n* (%)	163 (73.4%)	164 (73.9%)	0.914
Obesity			0.081
Yes, *n* (%)	42 (18.9%)	54 (24.3%)	
No, *n* (%)	173 (77.9%)	146 (65.8%)	
Missing, *n* (%)	7 (3.2%)	22 (9.9%)	
Smoking			0.089
Yes, *n* (%)	81 (36.5%)	73 (32.9%)	
No, *n* (%)	112 (50.4%)	143 (64.4%)	
Missing, *n* (%)	29 (13.1%)	6 (2.7%)	
Drinking			0.065
Yes, *n* (%)	71 (31.9%)	61 (27.5%)	
No, *n* (%)	122 (55.0%)	155 (69.8%)	
Missing, *n* (%)	29 (13.1%)	6 (2.7%)	
Hypertension			<0.001
Yes, *n* (%)	93 (41.9%)	176 (79.3%)	
No, *n* (%)	129 (58.1%)	46 (20.7%)	
Diabetes			<0.001
Yes, *n* (%)	23 (10.4%)	82 (36.9%)	
No, *n* (%)	199 (89.6%)	126 (56.8%)	
Missing, *n* (%)	—	14 (6.3%)	
Dyslipidemia			0.426
Yes, *n* (%)	100 (45.1%)	105 (47.3%)	
No, *n* (%)	121 (54.5%)	109 (49.1%)	
Missing, *n* (%)	1 (0.4%)	8 (3.6%)	
WBC (×10^9^/L)	—	11.26 ± 4.46	—
CRP (mg/L)	—	77.00 ± 60.88	—
D-dimer (*μ*g/mL)	—	4.65 ± 4.49	—
Max. aortic diameter (cm)	—	4.48 ± 0.95	—
Serum *TLR4* levels (ng/mL)	—	13.31 ± 6.74	—

^a^Demographic and clinical data for 222 AAD patients and 222 controls were collected from the medical records, and maximal aortic diameters of all cases were assessed by CTA. And serum *TLR4* levels in a total of 64 AAD subjects were obtained from our previously published data [18].

**Table 2:** The association of *TLR4* polymorphisms with the risk of AAD.

	NCBI Ref	Control	AAD	*P*(*P*_corr_)	OR (95% CI)
**rs10759932**	*n* = 28	*n* = 222	*n* = 222		
TT	13 (46.4%)	93 (41.9%)	106 (48.4%)		
TC	12 (42.9%)	95 (42.8%)	94 (42.9%)	0.297	0.759 (0.453-1.274)
CC	3 (10.7%)	34 (15.3%)	19 (8.7%)	**0.036 (0.072)**	**0.393 (0.164-0.939)**
CC+TC vs. TT				0.112	0.673 (0.412-1.098)
CC vs. TC+TT				**0.045 (0.090)**	**0.439 (0.196-0.984)**
*P*_HWE_		0.239	0.775		
**rs11536889**	*n* = 84	*n* = 222	*n* = 222		
GG	48 (57.1%)	154 (70.3%)	148 (66.7%)		
GC	31 (36.9%)	57 (26.0%)	58 (26.1%)	0.934	1.023 (0.590-1.775)
CC	5 (6.0%)	8 (3.7%)	16 (7.2%)	0.598	1.351 (0.441-4.135)
CC+GC vs. GG				0.818	1.063 (0.634-1.780)
CC vs. GC+GG				0.615	1.327 (0.441-3.995)
*P*_HWE_		0.351	0.004		

*P* for association was adjusted by age, sex, obesity, smoking, drinking, hypertension, diabetes, and dyslipidemia. NCBI Ref: reference frequencies of these SNPs in the Asian population (NCBI database). *P*_corr_: *P* values after Bonferroni correction. The results are in bold if *P* < 0.05.

**Table 3:** Association of *TLR4* polymorphisms with the risk of AAD stratified by age and sex.

	Genotypes	Control	AAD	*P*(*P*_corr_)	OR (95% CI)
**rs10759932**					
*Age*					
>55y	TT	50 (42.4%)	61 (52.1%)		
	TC	46 (39.0%)	45 (38.5%)	0.464	0.786 (0.413-1.497)
	CC	22 (8.6%)	11 (9.4%)	0.075	0.418 (0.160-1.093)
	CC+TC vs. TT			0.153	0.644 (0.353-1.177)
	CC vs. TC+TT			0.097	0.472 (0.195-1.144)
≤55y	TT	43 (41.3%)	45 (44.1%)		
	TC	49 (47.1%)	49 (48.0%)	0.598	0.787 (0.323-1.917)
	CC	12 (11.5%)	8 (7.8%)	0.240	0.275 (0.032-2.371)
	CC+TC vs. TT			0.482	0.731 (0.306-1.749)
	CC vs. TC+TT			0.315	0.355 (0.047-2.682)
*Sex*					
Male	TT	66 (40.5%)	67 (41.4%)		
	TC	70 (42.9%)	83 (51.2%)	0.560	1.198 (0.652-2.198)
	CC	27 (16.6%)	12 (7.4%)	0.061	0.373 (0.133-1.049)
	CC+TC vs. TT			0.935	0.976 (0.549-1.737)
	CC vs. TC+TT			**0.026 (0.052)**	**0.343 (0.133-0.882)**
Female	TT	27 (45.7%)	39 (68.4%)		
	TC	25 (42.4%)	11 (19.3%)	**0.015 (0.030)**	**0.231 (0.071-0.752)**
	CC	7 (11.9%)	7 (12.3%)	0.253	0.365 (0.065-2.054)
	CC+TC vs. TT			**0.010 (0.020)**	**0.241 (0.082-0.707)**
	CC vs. TC+TT			0.480	0.550 (0.105-2.890)
**rs11536889**					
*Age*					
>55y	GG	83 (70.9%)	78 (66.7%)		
	GC	30 (25.6%)	29 (24.8%)	0.935	1.029 (0.518-2.044)
	CC	4 (3.4%)	10 (8.5%)	0.436	1.807 (0.408-8.009)
	CC+GC vs. GG			0.702	1.134 (0.596-2.159)
	CC vs. GC+GG			0.423	1.831 (0.417-8.031)
≤55y	GG	71 (69.6%)	70 (66.7%)		
	GC	27 (26.5%)	29 (27.6%)	0.907	1.064 (0.379-2.983)
	CC	4 (3.9%)	6 (5.7%)	0.962	1.055 (0.116-9.595)
	CC+GC vs. GG			0.941	0.965 (0.367-2.532)
	CC vs. GC+GG			0.848	1.226 (0.153-9.824)
*Sex*					
Male	GG	114 (71.3%)	115 (70.1%)		
	GC	41 (25.6%)	40 (24.4%)	0.384	0.746 (0.386-1.442)
	CC	5 (3.1%)	9 (5.5%)	0.585	1.469 (0.369-5.839)
	CC+GC vs. GG			0.534	0.822 (0.444-1.524)
	CC vs. GC+GG			0.482	1.630 (0.418-6.360)
Female	GG	40 (67.8%)	33 (56.9%)		
	GC	16 (27.1%)	18 (31.0%)	**0.041 (0.082)**	**3.382 (1.051-10.885)**
	CC	3 (5.1%)	7 (12.1%)	0.590	1.729 (0.236-12.673)
	CC+GC vs. GG			**0.042 (0.084)**	**3.043 (1.041-8.900)**
	CC vs. GC+GG			0.786	1.313 (0.183-9.437)

*P* for association was adjusted by obesity, smoking, drinking, hypertension, diabetes, and dyslipidemia. *P*_corr_: *P* values after Bonferroni correction. The results are in bold if *P* < 0.05.

**Table 4:** The interaction effects between *TLR4* polymorphisms and risk factors in the susceptibility to AAD.

SNP genotypes	Number of subjects	Obesity^a^		Smoking^b^		Drinking^c^	Hypertension^d^		Dyslipidemia^e^		
		No	Yes	No	Yes	No	Yes	No	Yes	No	Yes
**rs10759932**											
CC+TC	No. of controls/cases	99/75	28/27	59/73	49/36	69/83	39/26	72/17	57/96	69/50	60/61
	OR (95% CI)	1.0 (ref.)	1.234 (0.651-2.337)	1.0 (ref.)	0.564 (0.319-0.997)	1.0 (ref.)	0.497 (0.268-0.922)	1.0 (ref.)	7.250 (3.662-14.353)	1.0 (ref.)	1.433 (0.825-2.488)
TT	No. of controls/cases	74/69	14/26	53/69	32/35	53/71	32/33	57/29	36/77	52/57	40/43
		1.035 (0.646-1.659)	1.952 (0.940-4.056)	0.954 (0.563-1.616)	0.837 (0.456-1.537)	1.065 (0.643-1.764)	0.716 (0.388-1.322)	1.657 (0.745-3.687)	7.812 (3.829-15.940)	1.360 (0.769-2.405)	1.377 (0.748-2.533)
	OR (95% CI)										
		*P*_interaction_ = 0.757, OR = 1.201 (0.377-3.830)		*P*_interaction_ = 0.304, OR = 1.684 (0.624-4.546)		*P*_interaction_ = 0.902, OR = 1.068 (0.376-3.035)	*P*_interaction_ = 0.585, OR = 0.750 (0.267-2.105)		*P*_interaction_ = 0.445, OR = 0.687 (0.263-1.798)		
CC	No. of controls/cases	24/12	10/4	16/11	12/8	19/13	9/6	20/2	14/17	20/8	14/11
	OR (95% CI)	1.0 (ref.)	0.768 (0.191-3.089)	1.0 (ref.)	0.889 (0.248-3.183)	1.0 (ref.)	0.768 (0.191-3.089)	1.0 (ref.)	10.045 (1.890-53.403)	1.0 (ref.)	3.667 (0.934-14.392)
TC+TT	No. of controls/cases	149/132	32/49	96/131	69/63	103/141	62/53	109/44	79/156	101/99	86/93
	OR (95% CI)	1.620 (0.741-3.545)	2.740 (1.141-6.579)	2.197 (0.927-5.209)	1.469 (0.604-3.572)	2.213 (1.005-4.872)	1.226 (0.531-2.831)	2.965 (0.647-13.597)	16.174 (3.635-71.956)	4.047 (1.300-12.603)	4.438 (1.419-13.883)
		*P*_interaction_ = 0.495, OR = 1.838 (0.320-10.550)		*P*_interaction_ = 0.699, OR = 0.729 (0.147-3.616)	*P*_interaction_ = 0.353, OR = 0.433 (0.074-2.533)				*P*_interaction_ = 0.665, OR = 0.664 (0.104-4.237)	*P*_interaction_ = 0.121, OR = 0.258 (0.047-1.428)	
**rs11536889**											
GG	No. of controls/cases	117/96	32/39	79/92	51/54	84/104	46/42	86/26	68/122	87/64	66/78
	OR (95% CI)	1.0 (ref.)	1.473 (0.833-2.605)	1.0 (ref.)	0.886 (0.532-1.473)	1.0 (ref.)	0.623 (0.363-1.068)	1.0 (ref.)	7.041 (3.785-13.097)	1.0 (ref.)	1.545 (0.938-2.548)
CC+GC	No. of controls/cases	53/50	10/15	33/51	28/19	38/51	23/19	41/20	24/54	33/45	32/27
	OR (95% CI)	1.049 (0.638-1.718)	1.561 (0.659-3.694)	1.333 (0.760-2.337)	0.583 (0.295-1.154)	1.061 (0.622-1.809)	0.604 (0.298-1.223)	1.618 (0.724-3.616)	7.596 (3.651-15.802)	1.500 (0.824-2.729)	1.034 (0.543-1.972)
		*P*_interaction_ = 0.870, OR = 0.114 (0.305-4.069)		*P*_interaction_ = 0.052, OR = 0.346 (0.118-1.011)	*P*_interaction_ = 0.273, OR = 0.531 (0.172-1.646)	*P*_interaction_ = 0.537, OR = 0.714 (0.245-2.078)				*P*_interaction_ = 0.095, OR = 0.413 (0.146-1.166)	
GC+GG	No. of controls/cases	164/136	41/51	106/134	78/67	116/143	68/58	124/42	87/164	113/101	97/97
	OR (95% CI)	1.0 (ref.)	1.463 (0.895-2.393)	1.0 (ref.)	0.664 (0.431-1.022)	1.0 (ref.)	0.603 (0.384-0.948)	1.0 (ref.)	5.873 (3.603-9.572)	1.0 (ref.)	1.071 (0.714-1.605)
CC	No. of controls/cases	6/10	1/3	6/9	1/6	6/12	1/3	3/4	5/12	7/8	1/8
	OR (95% CI)	1.805 (0.575-5.660)	3.384 (0.348-32.950)	1.053 (0.312-3.554)	4.386 (0.504-38.175)	1.571 (0.511-4.829)	1.746 (0.156-19.519)	1.613 (0.141-18.395)	8.065 (2.363-27.523)	1.143 (0.371-3.524)	7.838 (0.962-63.858)
		*P*_interaction_ = 0.999, OR = 0.999 (0.066-15.184)		*P*_interaction_ = 0.170, OR = 6.289 (0.456-86.754)	*P*_interaction_ = 0.515, OR = 2.700 (0.136-53.671)	*P*_interaction_ = 0.865, OR = 1.276 (0.076-21.291)				**P**_**interaction**_ = 0.038 **(0.076**^**#**^**),**OR = 15.229 **(1.156-200.621)**	

^a^ *P* for association was adjusted by age, sex, smoking, drinking, hypertension, diabetes, and dyslipidemia. ^b^*P* for association was adjusted by age, sex, obesity, drinking, hypertension, diabetes, and dyslipidemia. ^c^*P* for association was adjusted by age, sex, obesity, smoking, hypertension, diabetes, and dyslipidemia. ^d^*P* for association was adjusted by age, sex, obesity, smoking, drinking, diabetes, and dyslipidemia. ^e^*P* for association was adjusted by age, sex, obesity, smoking, drinking, hypertension, and diabetes. ^#^*P* value after Bonferroni correction. The results are in bold if *P* < 0.05.

**Table 5:** FRPR values for significant results on associations between *TLR4* polymorphisms and AAD risk.

Genotype	Number of controls/cases	OR (95% CI)	*P*	Statistical power^a^	Prior probability^b^				
					0.25	0.1	0.01	0.001	0.0001
**rs10759932**									
CC vs. TT (overall)	34/19 vs. 93/106	0.393 (0.164-0.939)	0.036	<0.001	0.995	0.998	0.999	1.000	1.000
CC vs. TC+TT (overall)	34/19 vs. 188/200	0.439 (0.196-0.984)	0.045	<0.001	0.996	0.998	0.999	1.000	1.000
CC vs. TC+TT (male)	27/12 vs. 136/150	0.343 (0.133-0.882)	0.026	<0.001	0.993	0.996	0.999	1.000	1.000
TC vs. TT (female)	25/11 vs. 27/39	0.231 (0.071-0.752)	0.015	<0.001	0.987	0.994	0.999	1.000	1.000
CC+TC vs. TT (female)	32/18 vs. 27/39	0.241 (0.082-0.707)	0.010	<0.001	0.981	0.991	0.999	1.000	1.000
**rs11536889**									
GC vs. GG (female)	16/18 vs. 40/33	3.382 (1.051-10.885)	0.041	0.706	**0.236**	**0.392**	0.854	0.983	0.998
CC+GC vs. GG (female)	19/25 vs. 40/33	3.043 (1.041-8.900)	0.042	0.705	**0.241**	**0.398**	0.857	0.984	0.998
CC vs. GC+GG (interaction with dyslipidemia)	1/8 vs. 113/101	15.229 (1.156-200.621)	0.038	0.897	**0.184**	**0.320**	0.811	0.977	0.998

^a^The statistical power was calculated using the number of observations, ORs, and *P* values in this table. ^b^The results are in bold if FPRP < 0.5.

**Table 6:** Association of *TLR4* polymorphisms with clinical parameters and serum *TLR4* levels in AAD patients.

Variables	rs10759932			rs11536889		
	TT (*n* = 106)	TC (*n* = 94)	CC (*n* = 19)	GG (*n* = 148)	GC (*n* = 58)	CC (*n* = 16)
WBC (×10^9^/L)	10.84 ± 4.35	11.63 ± 4.39	11.48 ± 5.43	11.06 ± 4.47	11.80 ± 3.94	11.07 ± 6.12
CRP (mg/L)	77.03 ± 65.71	76.57 ± 54.78	71.58 ± 63.35	79.51 ± 63.79	72.95 ± 55.34	69.51 ± 55.72
D-dimer (*μ*g/mL)	4.39 ± 4.24	4.68 ± 4.49	6.34 ± 6.17	4.76 ± 4.57	4.81 ± 4.66	2.95 ± 2.40
Max. aortic diameter (cm)	4.40 ± 0.86	4.55 ± 1.06	4.69 ± 0.76	4.40 ± 0.82	4.55 ± 1.12	4.96 ± 1.12^∗^
Serum *TLR4* levels (ng/mL)	14.83 ± 7.25	12.35 ± 5.56	9.78 ± 6.13^∗^	13.97 ± 7.69	13.07 ± 5.69	10.83 ± 3.56

^∗^ *P* < 0.05 vs. wild-type.

## Data Availability

The data used to support the findings of this study are available from the corresponding author upon request.
